# Phenotypic and Molecular Epidemiology of ESBL-, AmpC-, and Carbapenemase-Producing *Escherichia coli* in Northern and Eastern Europe

**DOI:** 10.3389/fmicb.2019.02465

**Published:** 2019-11-22

**Authors:** Epp Sepp, Reidar Andreson, Arta Balode, Anastasia Bilozor, Age Brauer, Svetlana Egorova, Kristi Huik, Marina Ivanova, Lidia Kaftyreva, Siiri Kõljalg, Triinu Kõressaar, Maria Makarova, Jolanta Miciuleviciene, Kristiine Pai, Maido Remm, Tiiu Rööp, Paul Naaber

**Affiliations:** ^1^Department of Microbiology, Institute of Biomedicine and Translational Medicine, University of Tartu, Tartu, Estonia; ^2^Department of Bioinformatics, Institute of Molecular and Cell Biology, University of Tartu, Tartu, Estonia; ^3^Department of Biology and Microbiology, Rīga Stradiņš University, Riga, Latvia; ^4^Department of Microbiology, Central Laboratory, East-Tallinn Central Hospital, Tallinn, Estonia; ^5^Department of Enteric Infections, St. Petersburg Pasteur Institute, Saint Petersburg, Russia; ^6^HIV Dynamics and Replication Program, National Cancer Institute, National Institutes of Health, Frederick, MD, United States; ^7^Department of Microbiology, Vilnius City Clinical Hospital, Vilnius, Lithuania; ^8^SYNLAB Eesti, Tallinn, Estonia

**Keywords:** *Escherichia coli*, whole genome sequencing, multilocus sequence typing, ESBL/AmpC/Carbapenemase genes epidemiology, Northern and Eastern Europe

## Abstract

Extended-spectrum beta-lactamases (ESBL) and AmpC producing-*Escherichia coli* have spread worldwide, but data about ESBL-producing-*E. coli* in the Northern and Eastern regions of Europe is scant. The aim of this study has been to describe the phenotypical and molecular epidemiology of different ESBL/AmpC/Carbapenemases genes in *E. coli* strains isolated from the Baltic States (Estonia, Latvia, and Lithuania), Norway and St. Petersburg (Russia), and to determine the predominant multilocus sequence type and single nucleotide polymorphisms diversity of *E. coli* isolates deduced by whole genome sequencing (WGS). A total of 10,780 clinical *E. coli* strains were screened for reduced sensitivity to third-generation cephalosporins. They were collected from 21 hospitals located in Estonia, Latvia, Lithuania, Norway and St. Petersburg during a 5 month period in 2012. The overall prevalence of ESBL/AmpC strains was 4.7% by phenotypical test and 3.9% by sequencing. We found more strains with the ESBL/AmpC phenotype and genotype in St. Petersburg and Latvia than other countries. Of phenotypic *E. coli* strains, 85% contained confirmed ESBL genes (including *bla*_CTX–M_, *bla*_TEM–__29_, *bla*_TEM–__71_), AmpC genes (*bla*_CMY–__59_, *bla*_ACT–__12__/__–__15__/__–__20_, *bla*_ESC–__6_, *bla*_FEC–__1_, *bla*_DHA–__1_), or carbapenemase genes (*bla*_NDM–__1_). *bla*_CTX–M–__1_, *bla*_CTX–M__–__14_ and *bla*_CTX–M–__15_ were found in all countries, but *bla*_CTX–M–__15_ prevalence was higher in Latvia than in St. Petersburg (Russia), Estonia, Norway and Lithuania. The dominating AmpC genes were *bla*_CMY–__59_ in the Baltic States and Norway, and *bla*_DHA–__1_ in St. Petersburg. *E. coli* strains belonged to 83 different sequence types, of which the most prevalent was ST131 (40%). In conclusion, we generally found low ESBL/AmpC/Carbapenemase prevalence in *E. coli* strains isolated in Northern/Eastern Europe. However, several inter-country differences in distribution of particular genes and multilocus sequence types were found.

## Introduction

Antimicrobial resistance is an emerging problem worldwide. Each year, 33,000 people die from an infection due to bacterial resistance to antibiotics in Europe. The burden of infections with bacterial resistance to antibiotics on the European population is comparable to that of influenza, tuberculosis and HIV/AIDS combined (Cassini et al., 2019). It has been estimated that by 2050, 10 million lives a year and a cumulative 100 trillion USD economic output are at risk worldwide due to the rise of drug resistant infections if we do not find proactive solutions to slow down drug resistance (O’Neill, 2016).

Resistance of Gram-positive bacteria is generally stable or even decreasing in Europe, whereas resistance to Gram-negative bacteria (such as *Enterobacterales*) has an increasing trend in several European countries (EARS-Net, 2018).

One of the important resistance mechanisms of *Enterobacterales*, including *Escherichia coli*, is the production of extended-spectrum beta-lactamases (ESBLs), AmpC cephalosporinases and carbapenemases. ESBLs include mostly CTX-M, SHV, and TEM enzymes; AmpCs CMY, ACT, and DHA; and carbapenemases KPC, NDM, OXA-48 (Bush and Jacoby, 2010; Bush, 2018).

Prevalence of these beta-lactamases has been increasing all over the world, including European countries (Bevan et al., 2017). Data from the European Antimicrobial Resistance Surveillance Network shows that *E. coli* resistance to third-generation cephalosporins is lower in Northern and higher in the Southern and Eastern Europe (EARS-Net, 2018). The proportion of invasive *E. coli* isolates resistant to third-generation cephalosporins by EARS-Net 2017 report was 5.9% in Norway, 8.8% in Estonia, 16.8% in Lithuania, and 22% in Latvia (EARS-Net, 2018). Comparable data for Russia is absent. Data from WHO CAESAR 2016 report includes a limited number of strains from Western part of Russia and shows high proportion of invasive *E. coli* isolates resistant to third-generation cephalosporins (66%; World Health Organisation, 2016). However, genes responsible for resistance to the third-generation cephalosporins are not well described in this region (Edelstein et al., 2003; Naseer et al., 2009; Dumpis et al., 2010; Seputiene et al., 2010; Bevan et al., 2017).

The aim of this study has been to describe the prevalence and molecular mechanisms of resistance to third-generation cephalosporins in *E. coli* strains isolated from Estonia, Latvia, Lithuania, Norway, and St. Petersburg (Russia), and to determine the predominant multilocus sequence type and single nucleotide polymorphisms diversity of *E. coli* isolates deduced by whole genome sequencing (WGS).

## Materials and Methods

### Strain Collection

During a 5 month period in 2012, *E. coli* clinical isolates from 21 hospitals located in Estonia (*n* = 5), Latvia (*n* = 4), Lithuania (*n* = 3), Norway (*n* = 1), and St. Petersburg (Russia) (*n* = 8) were screened for reduced susceptibility to the third-generation of cephalosporins. Briefly, all clinically relevant materials (such as blood, pus, urine, and respiratory tract samples) taken in case of infection from any kind of patients (all ages, outpatients or hospitalized in any department) and sent to microbiology laboratories for culture were included in the study. Surveillance, environmental and clinically irrelevant samples were excluded. All non-duplicate *E. coli* isolates interpreted as a probable cause of infection were included to the study (excluding clinically irrelevant cases, such as probable colonization or contamination from indigenous microbiota), and tested for third-generation cephalosporins (at least for ceftazidime and ceftriaxone and/or cefotaxime). Duplicates were defined as the same species isolated from the same patients during the study period and showing the same resistance pattern. Thus, the first isolate from a patient was always included. In case of similar isolates that were found from different materials taken at the same time, invasive isolate was preferred (for example, an isolate from blood was collected instead of sputum). Written instructions for sampling and laboratory procedures, and laboratory materials (ESBL/AmpC, confirmations kits, quality control strains, and if needed antibiotic disc) were distributed to all participants. Beforehand our project’s country managers and technical coordinators participated in a training course to ensure similar handling of samples, performance of laboratory techniques and quality control.

### ESBL/AmpC Screening and Confirmation

Susceptibility testing used disk diffusion according to the guidelines of valid versions at the time of testing of European Committee of Antimicrobial Susceptibility Testing (EUCAST, as in the Baltic countries and Norway) or the Clinical and Laboratory Standards Institute (CLSI, used in St. Petersburg, Russia). Initial antimicrobial susceptibility testing was performed in each laboratory for local standard panel that includes mandatorily ceftazidime and ceftriaxone and/or cefotaxime. In *E. coli* isolates with reduced susceptibility to third-generation cephalosporins, ESBL and AmpC cephalosporinases production was confirmed in a local laboratory with a ESBL + AmpC confirmation kit (Rosco Diagnostica, Taastrup, Denmark) provided by the project coordinator. *E. coli* isolates with the ESBL/AmpC phenotype were stored and sent to Estonian reference center (Human Microbiota Biobank, University of Tartu, Tartu, Estonia)^1^ for deposition and future characterization. Identification of all strains was confirmed by MALDI-TOF MS (MALDI Biotyper, Bruker Daltonics GmbH, Germany).

### Bacterial DNA Extraction and Whole Genome Sequencing (WGS)

All *E. coli* isolates with ESBL/AmpC phenotype were sequenced. Briefly, DNA templates for sequencing were generated by growing cultures of *E. coli* isolates overnight on the Mueller-Hinton agar (Oxoid Limited, United Kingdom). The total bacteria DNA from the strains were extracted using QIAamp DNA Mini Kit (Qiagen, Germany).

Bacterial genomic DNA was quantified using the Qubit^®^ 2.0 Fluorometer (Invitrogen, Grand Island, NE, United States). 1 ng of sample DNA was processed for the sequencing libraries, using Illumina Nextera XT sample preparation kit (Illumina, San Diego, CA, United States). The DNA normalization step was skipped; instead, the final dsDNA libraries were quantified with the Qubit^®^ 2.0 Fluorometer and pooled in equimolar concentrations. The library pool was validated with the 2200 TapeStation (Agilent Technologies, Santa Clara, CA, United States) measurements) and qPCR used the Kapa Library Quantification Kit (Kapa Biosystems, Woburn, MA, United States) to optimize cluster generation. A total of 96 bacterial genomic libraries were sequenced with 2 × 101 bp paired-end (PE) reads on the HiSeq 2500 rapid-run flow cell (Illumina, San Diego, CA, United States). Demultiplexing was done with CASAVA 1.8.2. (Illumina, San Diego, CA, United States), allowing 1 mismatch in index reads.

All sequenced genomes were assembled *de novo* with assembler Velvet version 1.2 (Zerbino and Birney, 2008). Before assembly, all reads with low quality were removed after quality control with fastq_quality_trimmer (with parameter values –l 40, -t 30) and fastq_quality_filter (-q 25 –p 90) from FASTX-Toolkit^2^. Velvet was run with different parameter values (-max_gap_count -max_divergence -cov_cutoff -ins_length -min_pair_count) until the best match of *E. coli* MLST genes was retrieved.

### Finding Beta-Lactamase Genes From Assembled Genomes

Beta-lactamase genes were retrieved from the Comprehensive Antibiotic Resistance Database [CARD database; (McArthur et al., 2013)]. Thereafter, the sequences were searched with BLAST (identity cut-off 90% and alignment length 90% of shortest sequence) from assembled genomes. The assembled contigs were considered to originate from either the plasmid genome or the chromosomal genome based on a BLAST search. We used complete plasmid genomes and complete chromosomal genomes of *E. coli* from NCBI genomes database for the BLAST search (the best match based on BLAST Score and *E*-value are used for deciding the origin of a contig).

### Multi-Locus Sequence Typing (MLST)

For accurate multi-locus sequence typing of assembled *E. coli* genomes, a dedicated MLST tool was used, created by Torsten Seemann^3^, that calculates the MLST profile based on a BLAST (Altschul et al., 1997) alignment of the input sequence file and the specified allele set. Public *E. coli* database (Achtman scheme) for molecular typing was downloaded (with given date: 11.06.2019) from PubMLST^4^. Raw reads from isolates with undetermined MLST types were submitted to Enterobase, which assigned five new sequence types (9656, 9692, 9693, 9694, and 9696). In order to visualize evolutionary relationships between bacterial strains, we used PHYLOViZ 2.0a (Nascimento et al., 2017) that generates complete minimum spanning trees with goeBURST Full MST algorithm.

### Core Genome Analysis

Parsnp program from Harvest suite (Treangen et al., 2014) was run to create core genome alignment. The alignment was used to calculate the maximum likelihood phylogenetic tree, with RaxML under GTR-GAMMA model and with 100 bootstrap replicates (Stamatakis, 2014).

### SNP Analysis of ST131 Isolates

ST131 isolates were aligned with Parsnp using *Escherichia coli* EC958 as a reference ST131 strain (Forde et al., 2014). Core genome SNPs from the alignment were extracted with harvest-tools and pairwise SNP distances were used for UPGMA tree calculation conducted in MEGA7 (Kumar et al., 2016). Phylogenetic trees were visualized using iTOL (Letunic and Bork, 2019).

### Statistical Analysis

Statistical analysis used Past 3.22^5^. The prevalence of strains, genes, ST and clones were compared by Chi-squared test or Fisher’s exact test; *p* < 0.05 was considered statistically significant.

## Results

### Phenotypic and Genotypic Epidemiology of ESBL/AmpC/Carbapenemases Producing *E. coli* Strains

A total of 10,780 consecutive *E. coli* isolates from Estonia, Latvia, Lithuania, Norway and St. Petersburg (Russia) were screened for reduced sensitivity to third-generation cephalosporins. Of these, 5,486 (51%) were recovered from stationary patients and 5,294 (49%) from outpatients. A total of 508 (4.7%) *E. coli* strains showed ESBL/AmpC phenotype. Significant inter-country differences were found regarding the prevalence of *E. coli* showing ESBL/AmpC phenotype (Table 1).

**TABLE 1:** Epidemiology of ESBL/AmpC phenotypes, *bla* genes and confirmed ESBL, AmpC, Carbapenemases (Carba) associated genes in *E. coli* strains from different countries.

**Country (screened strains)**	**ESBL/AmpC phenotype**	**Any *bla* gene^∗^**	**Any confirmed ESBL/AmpC/Carba^∗∗^**	**ESBL**	**AmpC**	**ESBL + AmpC**	**Carba + AmpC**
	
	**Number of strains (%)**						
Estonia (*n* = 4,144)	154(3)^1,2,3^	127(3)^8,9,10^	94(2)^15,16^	79(1)^21,22^	13 (0.3)	2 (0.04)	
Latvia (*n* = 1,175)	111(9)^1,4,5^	110(9)^8,11,12^	107(9)^15,17,18^	97(8)^21,23,24^	8(0)^27^	1 (0.09)	1 (0.09)
Lithuania (*n* = 1,329)	35(2)^4,6^	31(2)^11,13^	29(2)^17,19^	22(1)^23,25^	5 (0.4)	2 (0.2)	
Norway (*n* = 3,006)	73(2)^2,5,7^	67(2)^9,12,14^	62(2)^18,20^	58(1)^24,26^	4(0)^27^		
St. Petersburg (*n* = 1,135)	135(11)^3,6,7^	133(11)^10,13,14^	129(11)^16,19,20^	105(9)^22,25,26^	5 (0.4)	19 (1.7)	
Total (*n* = 10,780)	508 (4.7)	468 (4.3)	421 (3.9)	360 (3.3)	35 (0.3)	24 (0.2)	1 (0.09)

*^1,3,4,5,6,7,10,11,12,13,14,15,16,17,18,19,20,21,22,23,24,25,26^*p* ≤ 0.001; ^2,8,27^*p* ≤ 0.01; ^9^*p* ≤ 0.05. ^∗^Including also TEM and SHV other than 2be Bush-Jacoby functional group and *bla* genes without well characterized function; ^∗∗^ Including only well characterized ESBL/AmpC/carbapenemases associated *bla* genes belonging to 1, 2be, 2ber, and 3a Bush-Jacoby functional groups.*

From 508 sequenced strains in total, 494 gave accepted sequence quality and could be analyzed. At least one *bla* (beta-lactamase) gene (including TEM and SHV other than 2be Bush-Jacoby functional group and *bla* genes without well characterized function) was found in 468 strains of the 10,780 screened isolates (4.3%, Table 1). Of the phenotypic ESBL/AmpC producing strains, 468 out of 494 (94.7%) harbored one of the bla genes: *bla*_CTX–M_ (*n* = 383; 77.5%); *bla*_SHV_ (*n* = 21; 4%); *bla*_TEM_ (*n* = 271; 54.9%); *bla*_CMY_ (*n* = 36; 7.9%); *bla*_ACT_ (*n* = 3; 0.6%); *bla*_ESC–__6_ (*n* = 2; 0.4%); *bla*_FEC–__1_ (*n* = 7; 1.4%); *bla*_DHA_ (*n* = 12; 2.4%); *bla*_OXA_ (*n* = 192; 38.9%); *bla*_NDM_ (*n* = 1; 0.2%).

Of 494 resistant strains, 421 (85%) carried any ESBL/AmpC/Carbapenemases encoding genes (Clinic, 2015). In remaining isolates (*n* = 74; 15%) no known ESBL/AmpC/Carbapenemases genes were found. Sequencing showed the highest percentage of *E. coli* strains with the ESBL genotype in St. Petersburg and Latvia compared to Estonia, Norway and Lithuania (Table 1).

Prevalence of ESBL (including *bla*_CTX–M_, *bla*_TEM–__29_, *bla*_TEM–__71_), AmpC (*bla*_CMY–__59_, *bla*_ACT–__12__/__–__15__/__–__20_, *bla*_ESC–__6_, *bla*_FEC__–__1_, *bla*_DHA–__1_), carbapenemases (*bla*_NDM–__1_) groups and their combinations by countries can be found in Table 2 and Supplementary Figure S1. In 393 (93%) strains only one ESBL or AmpC was found and in 28 (7%) different genes combinations were detected. The most common combination was *bla*_CTX–M–__14_ together with *bla*_DHA–__1_ followed by *bla*_CTX–M–__15_ with *bla*_CMY–__59_ (Table 2).

**TABLE 2:** Prevalence of different ESBL/AmpC/Carbapenemase (Carba) genes and their combinations in *E. coli* strains from different countries.

	**ESBL/AmpC/Carba genes**	**Estonia**	**Latvia**	**Lithuania**	**Norway**	**St. Petersburg**	**TOTAL**
ESBL	*bla*_CTX–M–__1_	6	5	2	2	1	16
	*bla*_CTX–M–__2_					1	1
	*bla*_CTX–M–__3_	4	3	1		7	15
	*bla*_CTX–M–__5_	1					1
	*bla*_CTX–M–__9_				1		1
	*bla*_CTX–M–__14_	12	5	8	9	12	46
	*bla*_CTX–M–__15_	47	79	10	35	80	251
	*bla*_CTX–M–__24_	1	1		1		3
	*bla*_CTX–M–__27_	2	1		6		9
	*bla*_CTX–M–__32_	3	1				4
	*bla*_CTX–M–__55_				4	1	5
	*bla*_CTX–M–__88_	1					1
	*bla*_CTX–M–__111_		1				1
	*bla*_CTX–M–__116_	1					1
	*bla*_CTX–M–__136_		1				1
	*bla*_TEM–__29_	1					1
	*bla*_TEM–__71_			1			1
AmpC	*bla*_CMY–__59_	11	6	5	4	2	28
	*bla*_FEC–__1_	2	1			3	6
	*bla*_ESC–__6_		1				1
Combinations	*bla*_CTX–M–__14_, *bla*_CTX–M–__15_					3	3
	*bla*_CTX–M–__3_, *bla*_ACT–__12_			1			1
	*bla*_CTX–M–__14_, *bla*_DHA–__1_					12	12
	*bla*_CTX–M–__14_, *bla*_FEC–__1_					1	1
	*bla*_CTX–M–__15_, *bla*_ACT–__15_	1					1
	*bla*_CTX–M–__15_, *bla*_ACT–__20_	1					1
	*bla*_CTX–M–__15_, *bla*_CMY–__59_		1			5	6
	*bla*_CTX–M–__15_, *bla*_ESC–__6_					1	1
	*bla*_CTX–M–__24_, *bla*_CMY–__59_			1			1
	*bla*_NDM–__1_, *bla*_CMY–__59_		1				1

### Beta-Lactamases Genes

#### ESBL Genes

In total, 383 out of 468 (81.3%) *bla* gene-positive *E. coli* strains had CTX-M gene (Table 2). Among ESBL-positive strains, *bla*_CTX–M–__15_ was predominated (*n* = 263; 68.7%) followed by *bla*_CTX–M–__14_ (*n* = 62; 16.2%). Three isolates had 2 different CTX-M genes (*bla*_CTX–M–__15_ with *bla*_CTX–__M–__14_). In total, 80% of the CTX-M genes were plasmid-mediated and the remaining 20% were located on the chromosome. Chromosome localizations was found in the case of 6 genes: *bla*_CTX–M–__1_, *bla*_CTX–M–__3_, *bla*_CTX–M–__14_, *bla*_CTX–M–__15_, *bla*_CTX–M–__24__._

Although, *bla*_CTX–M–__15_ was the most common gene in all the countries, some inter-country differences were found. This gene was more common in Latvia than in St. Petersburg, Estonia, Norway and Lithuania (82.5 versus 71.8, 61.3, 60.3, and 41.7%; *p* ≤ 0.01, *p* ≤ 0.01, *p* ≤ 0.01; *p* ≤ 0.001, respectively). Also strains isolated from St. Petersburg had more *bla*_CTX–M–__15_ genes compared to Lithuanian strains (71.8 vs. 41.7%; *p* < 0.05). Lithuanian strains carried more *bla*_CTX–M–__14_ genes than Latvian strains (33.3 vs. 5.6%; *p* = 0.0005).

ESBL associated TEM genes were found only in 2 cases: *bla*_TEM–__29_ in Estonian and *bla*_TEM–__71_ in Lithuanian.

#### AmpC Genes

Out of 468 *E. coli* strains with any *bla* gene, 60 (12.8%) were harboring one of the AmpC type gene. All *bla*_ACT–__12__/__–__15__/__–__20_, *bla*_DHA–__1_, *bla*_FEC–__1_ genes were located in plasmid, *bla*_ESC–__6_ in chromosome and *bla*_CMY–__59_ in plasmid or chromosome.

The most prevalent AmpC-like genes were *bla*_CMY–__59_ (*n* = 36; 60%), *bla*_DHA–__1_ (*n* = 12; 20%), and *bla*_FEC–__1_ (*n* = 7; 11.7%) alone or in combination with other genes (Table 2).

#### Other Beta-Lactamase Genes

A total of 271 (57.9%) *E. coli* strains with any *bla* gene carried *bla*_TE__*M*_: *bla*_TEM–__1_, *bla*_TEM–__29_, *bla*_TEM–__71_, *bla*_TEM–__76_, *bla*_TEM–__98_, *bla*_TEM–__135_, *bla*_TEM–__150_, *bla*_TEM–__198_,and *bla*_TEM–__199__._ The most dominant strain was *bla*_TEM–__1_ (in 246 strains). Only, 21 (4.5%) *bla* gene-positive *E. coli* strains had *bla*_SHV–__188_ gene; however, the activity of SHV-188 enzyme has not been properly described. 192 (41%) *bla* gene-positive *E. coli* strains carried *bla*_OXA–__1_ and 1 strain *bla*_OXA–__7__._ However, enzymes coded by these genes are not real ESBLs. One carbapenemase plasmid-associated *bla*_NDM–__1_ gene was found in combination with *bla*_CMY–__59_ (Table 2).

### Genotypes of *Bla* Genes Containing *E. coli* Strains

**FIGURE 1:**
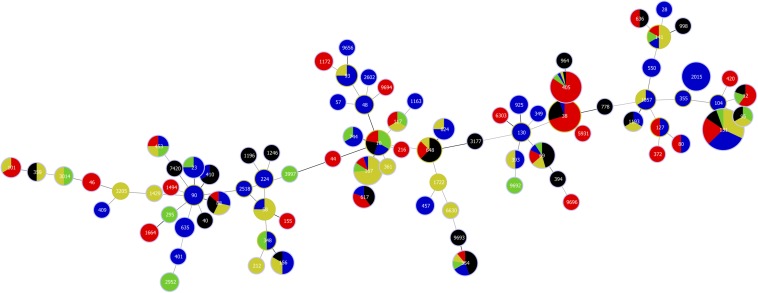
Distribution of *bla* genes containing *E. coli* multilocus sequence types (MLST) in Estonia (blue), Latvia (greenish yellow), Lithuania (green), Norway (black), and St. Petersburg (red).

We identified 83 different sequence types, the most prevalent being ST131 (*n* = 198; 40%), followed by ST38 (*n* = 37; 7.5%), ST405 (*n* = 32; 6.5%), ST167 (*n* = 19; 3.8%), and ST2015 (*n* = 18; 3.6%). The prevalence of other sequence types was <3%. When ST131 was common in all the countries, then ST167 was mainly found in Latvia (14/19; 73.7%), ST38 (25/37; 67.6%), and ST405 (27/32; 84.4%) in St. Petersburg and ST2015 (18/18; 100%) was found only in Estonia. Four sequence types were found in all countries: ST69, ST131, ST354, and ST405 (Figure 1 and Supplementary Figure S2).

ST131 was most common in Latvia (63/109; 57.8%), followed by Estonia (61/147; 41.5%), Lithuania (11/34; 32.4%), St. Petersburg (43/135; 31.9%), and Norway (20/6; 29%). In Latvia, the proportion of ST131 among ESBL strains was significantly higher than in St. Petersburg and Norway (*p* ≤ 0.001, *p* ≤ 0.001, respectively). Estonian strains belonged to more different sequence type groups when compared to Latvia, Lithuania and Norway (43 vs. 26, 19, 26; *p* ≤ 0.01, *p* ≤ 0.001, *p* ≤ 0.01, respectively). In St. Petersburg there were 30 different sequence types.

In ST131 strains, 7 different CTX-M genes were found: *bla*_CTX–M–__1_, *bla*_CTX–M–__3_, *bla*_CTX–M–__5_, *bla*_CTX–M–__14_, *bla*_CTX–M–__15_, *bla*_CTX–M–__27_, *bla*_CTX–M–__111_. ST38 strains carried mainly *bla*_CTX–M–__14_, and ST405 *bla*_CTX–M–__15_ genes; these sequence types were more frequent in St. Petersburg (ST38; 25/135; 18.5% and ST405 27/135; 20%) compared to other regions.

Analysis of single nucleotide polymorphisms (SNPs) in shared genome blocks revealed 18,888 SNPs present in the core genome of ST131 strains. Differences compared to reference EC958 ranged from 17 to 7,067 SNPs (Supplementary Figure S3). Four groups were detected among ST131 strains containing isolates from different countries, and having in-group average pairwise SNP distances of<20 (*n* = 40 strains; Supplementary Figure S4). SNP differences inside those four groups were 0–8.8 SNPs/Mbp of the ST131 core genome (∼3.4 Mbp). The average amount of SNPs between the groups was less than 110. One such group was also very close to reference EC958 strain. SNP tree of all *E. coli* strains is given in Supplementary Figure S2.

## Discussion

This study describes the phenotypic and molecular epidemiology of *E. coli* strains with reduced susceptibility to third-generation cephalosporins in Northern and Eastern Europe by screening of more than 10,000 *E. coli* strains. The overall prevalence of ESBL/AmpC strains was 4.7% by phenotypical test and 3.9% by sequencing. We found more strains with the ESBL/AmpC phenotype and genotype in St. Petersburg and Latvia than in other countries. According to our knowledge this is the first study analyzing beta-lactamases epidemiology of *E. coli* using WGS and describing in detail resistance genes, distribution of MLST and SNP clones in this region.

Although several reports have been previously published, scope, methodology and data quality in these studies vary. Edelstein’s group investigated *E. coli* strains from Russia, and found that the prevalence of phenotypic ESBL-positive strains was close to 16%; however, prevalence figures in different institutions varied from 10 to 90% (Edelstein et al., 2003). WHO CAESAR study reports high prevalence (66%) of third-generation cephalosporins resistance in invasive *E. coli* strains in Russia, however the number of strains was small (World Health Organisation, 2016). This high variance might be dependent upon different antibiotic use policies in different Russian regions and hospitals. Our results also showed a relatively high prevalence of ESBL/AmpC phenotype in St. Petersburg area compared to other countries. However, it is impossible to draw final conclusions about the overall prevalence of ESBL/AmpC/Carbapenemases in Russia at large, since the strains were collected only from St. Petersburg region, and thus reflects the situation in only one city.

In a previous Eastern European study conducted between 2004-2010 that included 42 centers, the average ESBL phenotype prevalence among *E. coli* strains was 15.3%, with the highest prevalence in South-East European countries - Turkey (25.2%), Bulgaria (15.7%) and Romania (12.2%) - and lowest in Central and North-East European countries - Croatia (3.6%) Czechia (3.6%), Latvia (3.6%), Slovenia (2.7%), and Lithuania (1.8%) (Balode et al., 2013). In a similar following study done between 2011 and 2016, the *E. coli* ESBL phenotype average prevalence was higher when compared to the previous survey (20.1 vs. 15.3%) (Balode et al., 2013; Dowzicky and Chmelarova, 2018). However, in these studies, phenotypic tests detecting only ESBL (not AmpC) were used. In countries of Western and Northern Europe, a prevalence of ESBL producing bacteria was low in the Netherlands (6.1%), Germany (7.7%), Sweden (2–4%), and Norway (1.5%) (Brolund et al., 2014; Soraas et al., 2014; Zhou et al., 2017).

When comparing ESBL/AmpC prevalence in different studies several aspects should be taken into account. Different methods and criteria have been used in different studies such as decreased sensitivity to third-generation cephalosporins as an indicator of ESBL; phenotypic confirmation test for ESBL alone or ESBL combined with AmpC. In studies where molecular methods were applied, different approaches have been used: searching only for CTX-M types or including also TEM, SHV and AmpC type genes.

Besides differences in detection methodologies several other factors might influence the results and potential cause over as well as underestimation of ESBL/AmpC prevalence and resistance percentages. One such factor is the use of different sampling practices in different institutions noted also by EARS-Net as a factor that should be taken into account in interpreting inter-country differences (EARS-Net, 2018). A similar limitation is present in all studies using clinical strains from routine cultures, including our study.

We found more strains with ESBL/AmpC phenotype than strains with known ESBL/AmpC gene. Several reasons can cause this: other mechanisms such as possible hyperproducers of intrinsic (chromosomal) cephalosporinase combined or not with alteration in porin channels can lead to resistance to third-generation cephalosporins; *bla* genes databases are not complete - we probably don’t know all ESBL/AmpC genes or not all are submitted to databases, furthermore these genes are changing and new variants may not be recognized; we found in our strains several genes (such as SHV and TEM variants) without information about their belonging to particular Bush-Jacoby functional group (ESBL/AmpC or not). Only well described ESBL/AmpC genes were included in this study.

The most common ESBL genes in our study were *bla*_CTX–M–__15_ and *bla*_CTX–M–__14_. These enzymes have been reported throughout Asia, Africa, Europe, America and Australia (Livermore et al., 2007; Sidjabat et al., 2010; Iroha et al., 2012; Chen et al., 2014; Pietsch et al., 2017). So far, the CTX-M-15 genotype appears to be the most prevalent in all continents, and our findings are in accordance with previous reports (Sidjabat et al., 2010; Canton et al., 2012; Iroha et al., 2012; Voets et al., 2012; Brolund et al., 2014; Chen et al., 2014; Bevan et al., 2017; Jorgensen et al., 2017; Pietsch et al., 2017). CTX-M-15 dominates in Germany and the Netherlands, but more recent studies show an increased proportion of CTX-M-1 compared to CTX-M-14 (Voets et al., 2012; Pietsch et al., 2017). CTX-M-14 has been found to be less prevalent in most countries with some exceptions [China, South-East Asia, South Korea, Japan, and Spain; (Onnberg et al., 2011; Copur Cicek et al., 2013; Helldal et al., 2013; Bevan et al., 2017)]. Although we found CTX-M-14 in all investigated countries it was less prevalent than CTX-M-15. We found in few cases (0.7%) the combination of CTX-M-14 and CTX-15. In some regions this combination was frequently reported (Park et al., 2012). Increasing prevalence of CTX-M-27 has been reported worldwide. This genotype is a single nucleotide variant of CTX-M-14 showing higher MIC to ceftazidime and therefore use of ceftazidime would theoretically select it (Bevan et al., 2017). We found only a few CTX-M-27 strains from Estonia, Latvia and Norway. As in previous studies we found the majority of *bla*_CTX–M_ in plasmids and a minority (20%) in chromosome. However, frequency of chromosomal location of *bla*_CTX–M_ (mainly *bla*_CTX–M–__14_ and *bla*_CTX–M–__15_) varies in different regions and studies from<5% in some European countries to 27% in recent Japanese study (Rodríguez et al., 2014; Hamamoto and Hirai, 2019).

In previous studies AmpC prevalence in *E. coli* was usually low, however in some regions prevalence up to 9% has been reported (Pascual et al., 2016; Zhou et al., 2017; Kazemian et al., 2019; Ribeiro et al., 2019). In our study AmpC prevalence was<1% except for in St. Petersburg (2.1%). In the previous studies, CMY-2 was usually the most common AmpC, however DHA-1 has been reported as dominant in some studies (Brolund et al., 2014; Soraas et al., 2014; Pascual et al., 2016; Kazemian et al., 2019; Ribeiro et al., 2019). In our study *bla*_CMY–__59_ was dominating in the Baltic States and Norway but *bla*_DHA–__1_ in St. Petersburg. There are only a few reports about finding *bla*_CMY–__59_ in clinical strains (Roy et al., 2011; Ranjbar et al., 2013). In some AmpC epidemiology studies, common predominance of “CMY-2 like” genes has been reported without exact gene determination that makes it difficult to compare our data with others (den Drijver et al., 2018; Pietsch et al., 2018).

Only one NDM-1-producing *E. coli* was found during our study. Carbapenem resistance is still rare among *E. coli* strains in Europe (0–1.6%) and *bla*_OXA–__48_ is the most commonly observed carbapenemase. At the same time carbapenem resistant *K. pneumoniae* is more common in Europe (0–64.7%) with *bla*_*KPC*_ and *bla*_OXA–__48_ predominance (Grundmann et al., 2017; EARS-Net, 2018). However, outbreak of NDM-1-producing *K. pneumoniae* has been reported in St. Petersburg (Pavelkovich et al., 2014). No co-production of NDM-1 and CMY-39 has been reported previously. Prevalence of carbapenemases among other Enterobacterales is probably rare. In our study in Northern and Eastern Europe (2015, including nine countries) only one *bla*_OXA–__48_ was found in 88 Enterobacterales strains (other than *K. pneumoniae*) with reduced susceptibility to carbapenems; in the same settings ca 50% of *K. pneumoniae* strains with reduced susceptibility to carbapenems (*n* = 171) harbored carbapenemase gene (our unpublished data).

In sequenced strains presence of TEM, SHV or OXA genes was common. However, only a few (< 1%) TEM genes were real ESBL/AmpC encoding genes. In other cases these genes were not associated with ESBL/AmpC phenotype or their belonging to Bush-Jacoby functional group is unknown. Thus, detection of TEM, SHV or OXA genes without sequencing have no value in ESBL epidemiology studies.

ST131, which belongs to the highly virulent phylogenetic group B-group, is prevalent worldwide (Sidjabat et al., 2010; Voets et al., 2012; Brolund et al., 2014; Bevan et al., 2017; Jorgensen et al., 2017; Pietsch et al., 2017; Zhou et al., 2017; Chong et al., 2018). According to other studies, ST131 usually contains different *bla*_CTX–M_, the most common being *bla*_CTX–M–__15_, followed by *bla*_CTX–M–__14_, and *bla*_CTX–M–__27_ as also found in our region (Sidjabat et al., 2010; Brolund et al., 2014; Chen et al., 2014; Bevan et al., 2017; Chong et al., 2018; Teunis et al., 2018).

ST131 was also the most common genotype in our study. More than 50% of the Latvian and over one-third of Estonian, Lithuania, and St. Petersburg’s *E. coli* strains belonged to this group. When applying SNP analysis to ST131 strains several clones with cross-border spreading were found.

In general, prevalence of ESBL, AmpC and Carbapenemases genes was low in investigated *E. coli* strains. However, several inter-country differences notably in distribution of particular genes, MLST groups and SNP clones, were described.

## Data Availability Statement

The datasets generated for this study can be found in the NCBI GenBank https://www.ncbi.nlm.nih.gov/bioproject/PRJNA528606.

## Ethics Statement

Approval was not required as per the local legislation. Institutions used only samples sent for routine diagnostics, no additional sampling being necessary. No patient data was used, and all strains were coded and processed anonymously (it is impossible to identify any patient by strain number).

## Author Contributions

ES: principal investigator, preparation of the manuscript. RA: bioinformatics. ArB: BEEP/BARN coordinator in Latvia, data and strain collection, critical reading of the manuscript and SNP analyses. AnB: principal investigator, preparation of manuscript, BEEP/BARN Estonian coordinator. AgB: SNP analyses. SE: BEEP/BARN coordinator in Russia, data and strain collection, critical reading of the manuscript. KH: molecular studies, critical reading of the manuscript. MI: BEEP/BARN international technical coordinator, data preparation of the manuscript. LK: BEEP/BARN coordinator in Russia, data and strain collection, critical reading of the manuscript. SK: ARMMD coordinator, preparation of the manuscript. TK: WGS data analyses. MM: BEEP/BARN coordinator in Russia, data and strain collection, critical reading of article. JM BEEP/BARN coordinator in Lithuania, data and strain collection, critical reading of the manuscript. KP: design of molecular studies and strains preparation. MR: WGS data analyses and preparation of the manuscript. TR: strains characterization and responsible for culture collection. PN: scientific coordinator and preparation of manuscript.

## Conflict of Interest

The authors declare that the research was conducted in the absence of any commercial or financial relationships that could be construed as a potential conflict of interest.

## Footnotes

1 http://eemb.ut.ee/eng/humb_english_introduction_list.php

2 http://hannonlab.cshl.edu/fastx_toolkit/index.html

3 https://github.com/tseemann/mlst

4 http://www.pubmlst.org/

5 http://folk.uio.no/ohammer/past/

## Supplementary Material

FIGURE S1 Prevalence of ESBL/AmpC/carbapenemases genes in *E. coli* strains isolated from Estonia, Latvia, Lithuania, Norway, and St. Petersburg.

FIGURE S2 Maximum likelihood phylogeny of the core genome of studied *E. coli* isolates calculated with RaxML. Top five most frequently observed sequence types are marked in color: ST131 (red), ST38 (yellow), ST405 (green), ST167 (blue), ST2015 (purple). Country codes (two first letters in strain codes): EE, Estonia; LV, Latvia; LT, Lithuania; NO, Norway; RU, St Petersburg (Russia). Isolates NOR5_51 and EETUKB199 were not used in further analysis because of the low assembly quality.

FIGURE S3 UPGMA tree based on core SNP distances among ST131 isolates. Country codes (two first letters in strain codes): EE, Estonia; LV, Latvia; LT, Lithuania; NO, Norway; RU, St Petersburg (Russia).

FIGURE S4 Higher resolution subpart of ST131 tree from Supplementary Figure S3. Groups containing isolates from different countries and average pairwise distances less than 20 SNPs inside the group are marked. Country codes (two first letters in strain codes): EE, Estonia; LV, Latvia; LT, Lithuania; NO, Norway; RU, St Petersburg (Russia).
