# Supplementary figures and images for: Phenotypic and Molecular Epidemiology of ESBL-, AmpC-, and Carbapenemase-Producing Escherichia coli in Northern and Eastern Europe
Source: Front Microbiol. 2019 Nov 22;10:2465. doi: 10.3389/fmicb.2019.02465 (PMC6882919; doi:10.3389/fmicb.2019.02465)

## Estonia

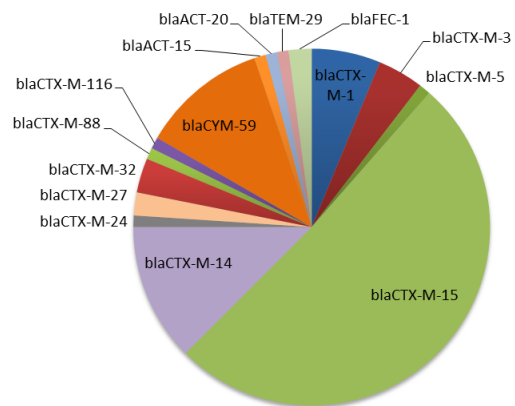

## Norway

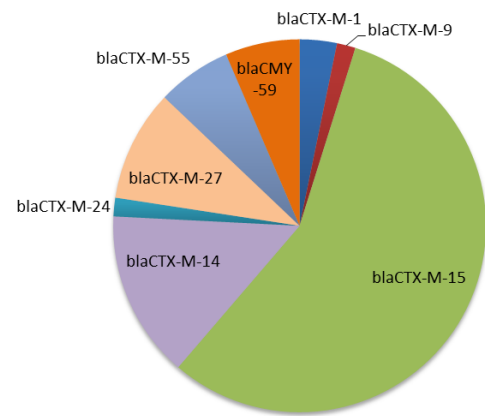

## Latvia

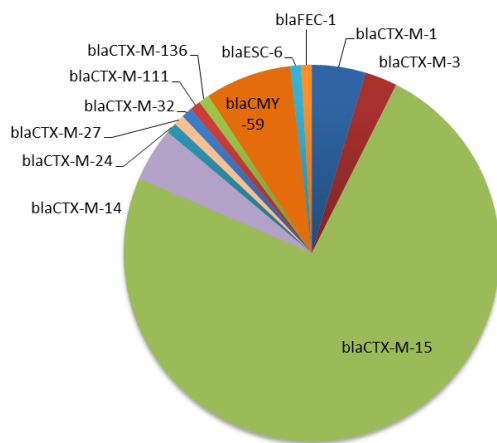

## St. Petersburg

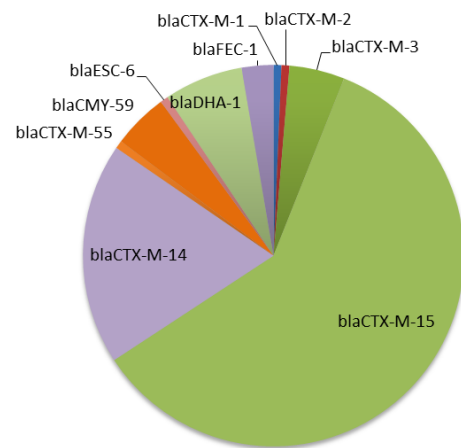

## Lithuania

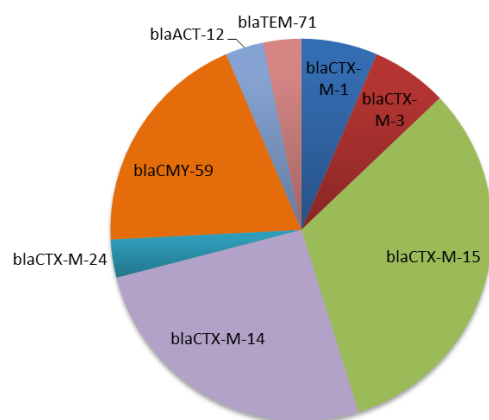

Supplement: FIGURE S1 — Prevalence of ESBL/AmpC/carbapenemases genes in E. coli strains isolated from Estonia, Latvia, Lithuania, Norway, and St. Petersburg. [file Data_Sheet_1.PDF]

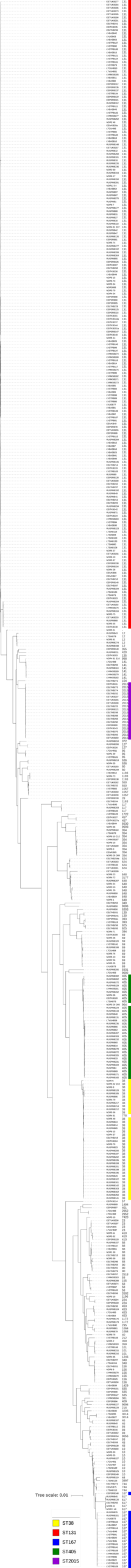

Supplement: FIGURE S2 — Maximum likelihood phylogeny of the core genome of studied E. coli isolates calculated with RaxML. Top five most frequently observed sequence types are marked in color: ST131 (red), ST38 (yellow), ST405 (green), ST167 (blue), ST2015 (purple). Country codes (two first letters in strain codes): EE, Estonia; LV, Latvia; LT, Lithuania; NO, Norway; RU, St Petersburg (Russia). Isolates NOR5_51 and EETUKB199 were not used in further analysis because of the low assembly quality. [file Data_Sheet_2.PDF]

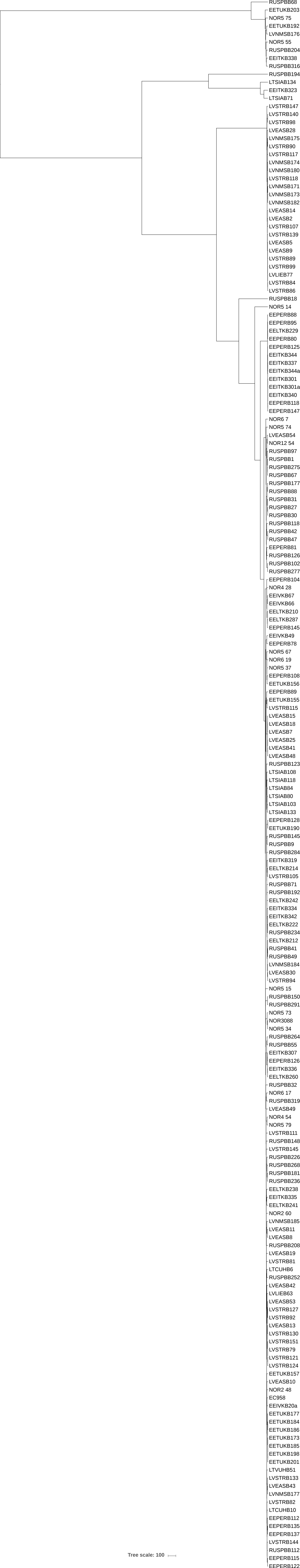

Supplement: FIGURE S3 — UPGMA tree based on core SNP distances among ST131 isolates. Country codes (two first letters in strain codes): EE, Estonia; LV, Latvia; LT, Lithuania; NO, Norway; RU, St Petersburg (Russia). [file Data_Sheet_3.PDF]

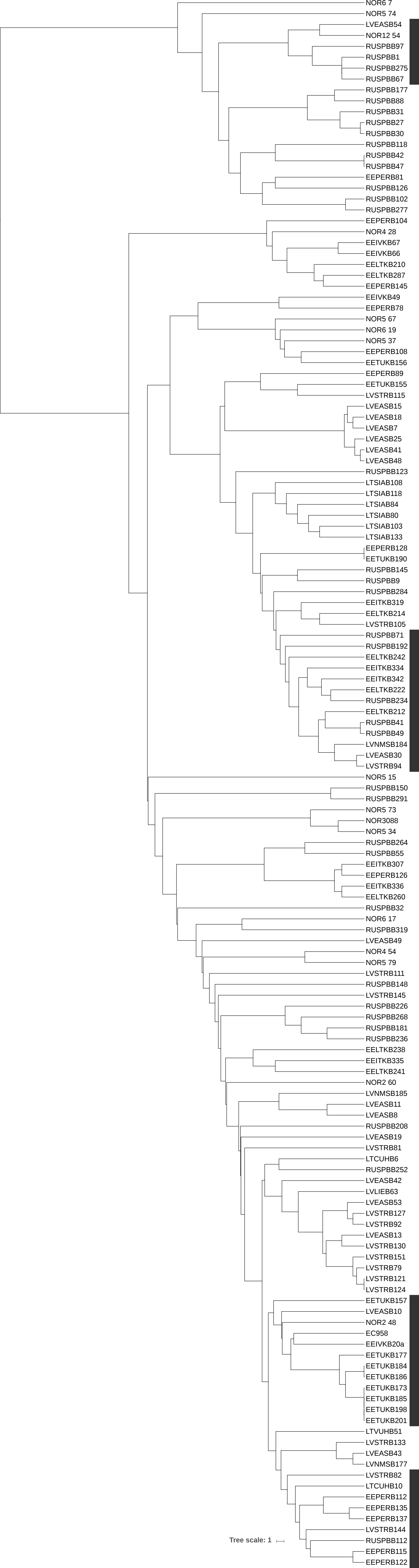

Supplement: FIGURE S4 — Higher resolution subpart of ST131 tree from Supplementary Figure S3. Groups containing isolates from different countries and average pairwise distances less than 20 SNPs inside the group are marked. Country codes (two first letters in strain codes): EE, Estonia; LV, Latvia; LT, Lithuania; NO, Norway; RU, St Petersburg (Russia). [file Data_Sheet_4.PDF]
